# Ultra-high rate of temperature increment from superparamagnetic nanoparticles for highly efficient hyperthermia

**DOI:** 10.1038/s41598-021-84424-1

**Published:** 2021-03-02

**Authors:** Jae-Hyeok Lee, Bosung Kim, Yongsub Kim, Sang-Koog Kim

**Affiliations:** National Creative Research Initiative Center for Spin Dynamics and Spin-Wave Devices, Nanospinics Laboratory, Research Institute of Advanced Materials, Department of Materials Science and Engineering, Seoul National University, Seoul, 151-744 South Korea

**Keywords:** Ferromagnetism, Magnetic properties and materials, Magnetic properties and materials, Nanoparticles

## Abstract

The magneto-thermal effect, which represents the conversion of magnetostatic energy to heat from magnetic materials, has been spotlighted for potential therapeutic usage in hyperthermia treatments. However, the realization of its potential has been challenged owing to the limited heating from the magnetic nanoparticles. Here, we explored a new-concept of magneto-thermal modality marked by low-power-driven, fast resonant spin-excitation followed by consequent energy dissipation, which concept has yet to be realized for current hyperthermia applications. We investigated the effect of spin resonance-mediated heat dissipation using superparamagnetic Fe_3_O_4_ nanoparticles and achieved an extraordinary initial temperature increment rate of more than 150 K/s, which is a significant increase in comparison to that for the conventional magnetic heat induction of nanoparticles. This work would offer highly efficient heat generation and precision wireless controllability for realization of magnetic-hyperthermia-based medical treatment.

## Introduction

Hyperthermia therapy is a type of medical treatment in which a target region of the body is exposed to higher-than-surrounding temperatures in order to kill targeted cancer cells^1^ or at least to make them more sensitive to other therapeutic means such as high gamma radiation and certain anticancer drugs^2^. Magnetic hyperthermia using magnetic nanoparticles^3,4^, with the advantage of its non-interactive penetration of magnetic field into biological systems, has become one of the leading methodologies among hyperthermia treatments such as photo-thermal therapy^5^, microwave irradiation^6^, ultrasound^7^, and laser-induced hyperthermia^8^. For magnetic hyperthermia, the heat required for apoptosis or thermo-ablation of tumor cells is known to be derived by Néel-Brownian motions and/or hysteresis loss of magnetic nanoparticles using several-hundred-kilohertz AC magnetic fields^9^. Over the last decade, there have been significant progresses in the development of magnetic hyperthermia to enhance AC-induction-heating capability: successful syntheses of FeCo nanoparticles^10^, core-shell^11^ or multi-core-shell^12^ iron-oxide particles, and Mg-doped^13^ iron-oxide particles of improved magnetocrystalline anisotropy and magnetic susceptibility. Although several successful clinical trials have been carried out on glioblastoma tumors^14,15^, there are still challenging obstacles to conventional magnetic hyperthermia. The relatively low heating rate of conventional magnetic hyperthermia typically with Fe_3_O_4_, for example, often requires a high concentration of nanoparticles to be injected into the human body, which not only would result in potential toxicity but also complicates monitoring of the progress of tumor response using medical imaging tools^16^. Also, it would be desirable to reach a target temperature instantaneously, but frequently observed delayed response time renders precise control of relevant temperature at/near targeted tumors difficult, leading consequently to undesired side effects such as overheating of surrounding normal tissues and/or non-uniform distribution of temperature near/in tumors. To overcome some of critical challenges of conventional hyperthermia, a quantum-leap approach through the state-of-the-art technology that could significantly enhance heating performance is critically important. Our earlier theoretical work^17,18^ suggested that the resonant spin-excitation and dissipation of a single nanosphere model allow for specific loss power (SLP) values of 2 or 3 orders of magnitude larger than those of conventional hyperthermia (100–1,000 W/g). In this study, we experimentally demonstrated the magneto-thermal effect of resonant spin-excitation and consequent dissipation of magnetic nanoparticles. The increase of the intrinsic temperature of nanoparticles associated with the energy conversion at resonance was measured directly from a heat source (i.e. magnetic particles) without any solution environment via the infrared (IR) thermographic method. The novel mechanism offers exceedingly high-efficiency ultrafast local heating and consequently exceptionally high rates of temperature increment by adjustment of controllable field parameters such as the frequency and strength of AC magnetic fields and pulse width as well as DC field strength.

## Results

### High-efficiency heat generation based on resonant spin-excitation and dissipation

**Figure 1:**
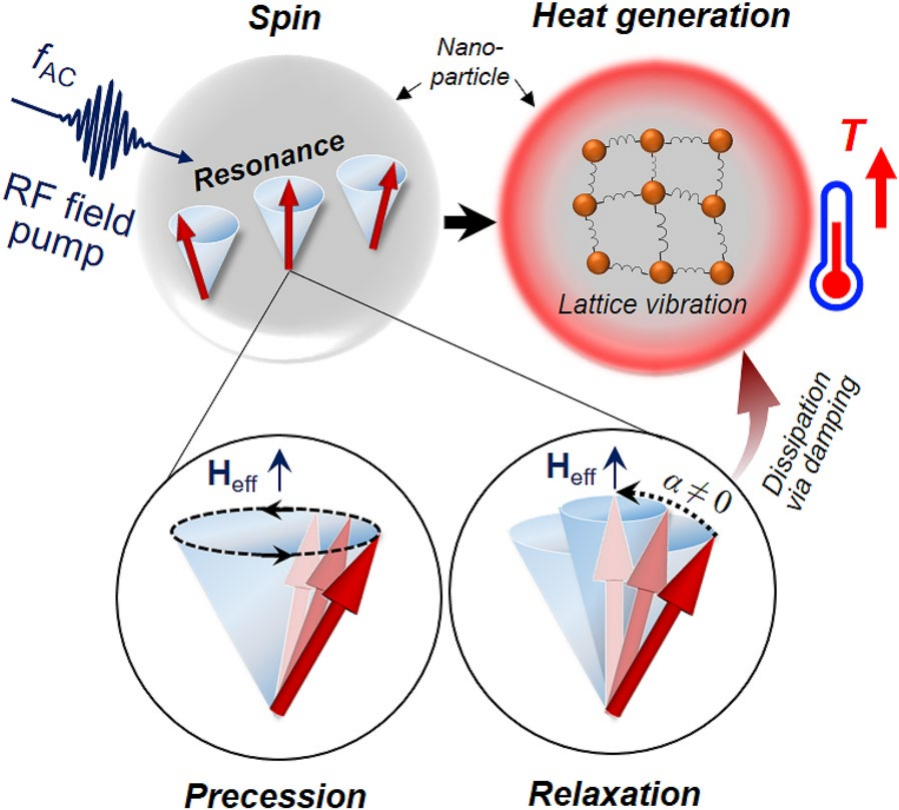
Resonant spin-excitation and relaxation dynamics for dissipative local heating. RF magnetic fields were applied to excite magnetic nanoparticles under an external DC magnetic field. When the RF field frequency was tuned to an intrinsic resonant frequency of the precession of individual magnetic moments (indicated by red arrows), the precession on a specific angle occurred around the internal effective field (see blue cones). Simultaneously, the precession of the magnetic moments started to lose a certain amount of absorbed magnetic energy owing to intrinsic damping, resulting in the reorientation of magnetizations in the DC field direction. The energy due to magnetic loss was dissipated in the form of lattice vibrations through the various spin–lattice interactions, thereby increasing the temperature of the magnetic nanoparticles.

Figure 1 shows the underlying mechanism of resonant spin-excitation and consequent dissipation in magnetic nanoparticles. When a DC magnetic field of sufficient strength is applied to magnetic particles, individual magnetic moments (spins) inside them are aligned in the direction of the DC magnetic field. Then, a microwave magnetic field applied to the particles can make the magnetization (**M**) precess around the direction of the effective field (**H**_eff_) inside the magnetic particles. This precessional motion of **M** can be effectively excited under its resonance condition, in which case the frequency of the microwave field is tuned to the intrinsic resonance frequency of the precession of **M**, as expressed by $f_{{\text{R}}} = \left( {{\gamma \mathord{\left/ {\vphantom {\gamma {2\pi }}} \right. \kern-\nulldelimiterspace} {2\pi }}} \right)H_{{{\text{eff}}}}$, with *γ* the gyromagnetic ratio. This entire dynamic motion of **M** is expressed by Landau-Lifshitz-Gilbert (LLG) equation $d{\mathbf{M}}/dt = - \gamma {\mathbf{M}} \times {\mathbf{H}}_{{{\text{eff}}}} + (\alpha /M_{{\text{S}}} ){\mathbf{M}} \times d{\mathbf{M}}/dt$, where the terms correspond to the precession of **M** and its phenomenological damping, respectively. The precession motion excited by the microwave field is purely dissipative via damping; thus, the energy of the magnetic system is converted to heat through nonlinear spin relaxations introduced by various interactions such as spin–orbit coupling, two-magnon scattering, and field inhomogeneity^19^. Since the time scale of spin dynamics is on the order of nano seconds^20^, ultra-fast time-scale local heating is possible, indeed faster than μs in principle, compared with that of Néel-Brownian relaxation mechanism^21^. In earlier work, we reported that the resonant spin dynamics of magnetic particles in the uniform magnetization state or vortex state according to particle size^17,18,22^ can theoretically be used as a novel means to make nanoparticles a local heat source of exceedingly high heating power. Here, in the present study, we experimentally demonstrated the thermal effect of resonant spin excitation and relaxation using Fe_3_O_4_ magnetic nanoparticles.

### Measurement of temperature increments from Fe_3_O_4_ nanoparticles

**Figure 2:**
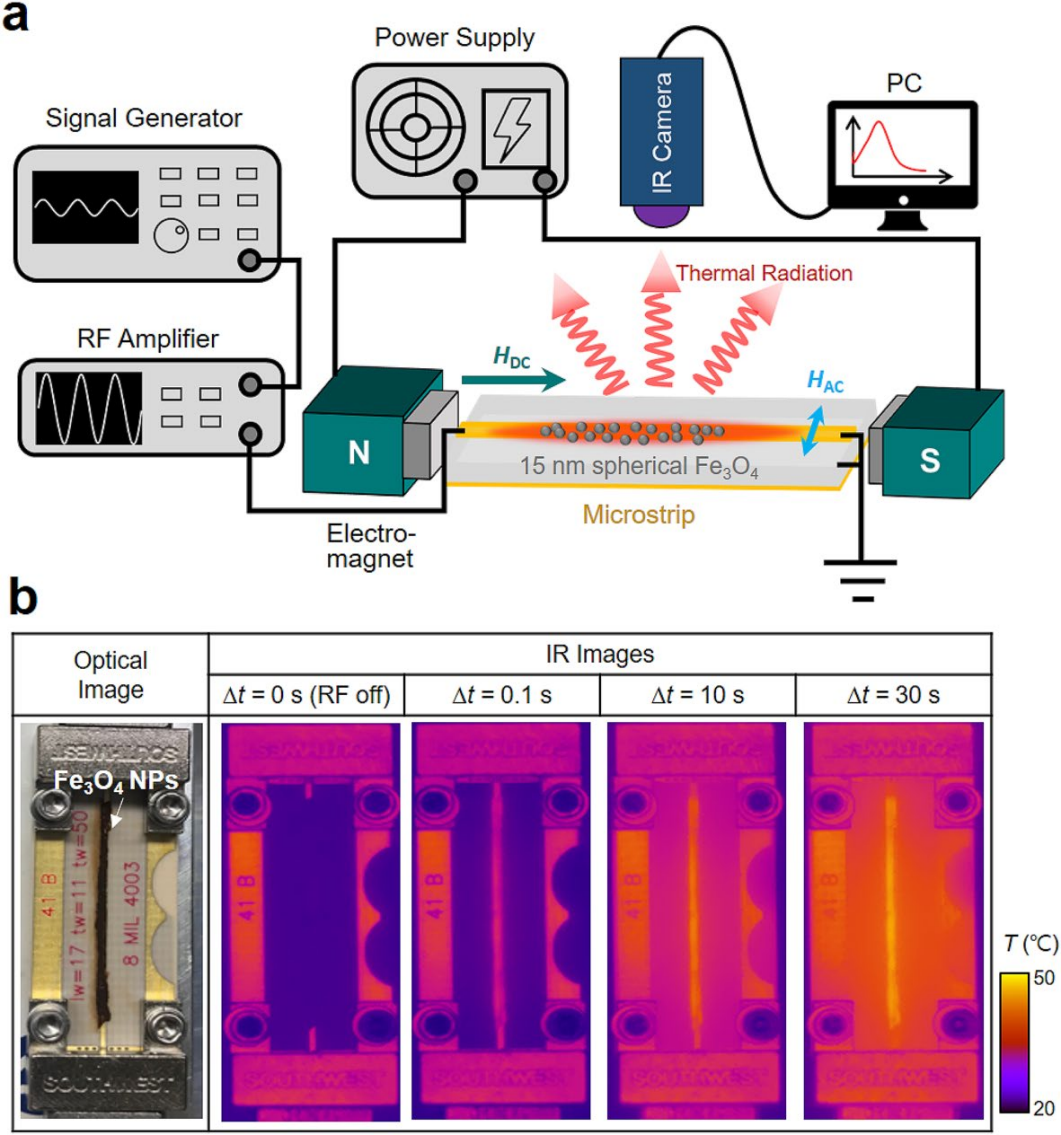
Experimental measurement of temperature increment from Fe_3_O_4_ nanoparticles via thermal radiation. (**a**) Experimental apparatus for measurements of temperature increment by resonant spin-excitation and dissipation in superparamagnetic, 15-nm-diameter Fe_3_O_4_ nanoparticles. In order to generate RF magnetic fields (by power pumping), RF currents of GHz frequencies were transmitted to the microstrip using a signal generator and an RF amplifier. A DC magnetic field was also applied on the axis of a microstrip line. The direction of the RF magnetic field was perpendicular to the DC field direction. The change of temperature of the particles was recorded by an IR camera through thermal radiation. A detailed explanation of the experimental procedure is given in Method. (**b**) Optical image (left) of sample stage and thermal IR images (right four) for different duration times ∆*t* of application of RF magnetic fields (i.e. ∆*t* = 0, 0.1, 10, and 30 s). The color bar on the right indicates the local temperature.

In order to investigate heat generation via the energy dissipation associated with the above-noted mechanism, we devised an apparatus for measurement of Fe_3_O_4_ particle temperature. The apparatus is composed mainly of a radio-frequency (RF) power pumping system to generate resonant spin-excitation and a thermal IR camera to directly measure the temperatures of 15 nm-size Fe_3_O_4_ particles without silica shells (for details on the synthesis of Fe_3_O_4_ nanoparticles, see Supplementary Section S1) via thermal-radiation detection means, as schematically represented in Fig. 2a (for details on the measurement system, see “Methods”). Figure 2b displays, as examples, the results of optical measurements. The first optical image shows the Fe_3_O_4_ nanoparticles placed on a sample stage, and on the right, four different IR images are shown. The second image shows the distribution of local temperatures without application of an RF field, while the third, fourth, and fifth images in the series show temperature increments from the Fe_3_O_4_ particles under application of the resonant RF field (*f*_AC_ = 3.0 GHz and *H*_AC_ = 2.37 Oe) for different duration times of 0.1, 10, and 30 s, respectively. In all of the cases, a DC field strength of *H*_DC_ = 750 Oe was applied for measurement of the IR images. In the RF-off IR image, the purple color in the middle corresponds to the room temperature of the magnetic particles. Immediately after applying the RF field, at Δ*t* = 0.1 s, the color of the Fe_3_O_4_ particles turns to red, as observed only in the region of the particles. For the longer duration time of Δ*t* = 10 s, the red color becomes yellow, indicating that the temperature increases further. For Δ*t* = 30 s, the yellow color becomes brighter, corresponding to a 45 ℃ particle temperature. The colors of the sample stage also turned out to be red and then yellow, because the sample stage was also heated to 34 ℃ through thermal-conduction-based heat transfer. These IR images clearly reveal that the temperature increment of Fe_3_O_4_ particles can be quantitatively measured via thermal radiation entailing heating of particles by resonant spin-excitation and the consequent continuous dissipation. The temperature measurements were carried out for different strengths, frequencies, and pulse widths of RF magnetic fields as well as DC field strengths.

**Figure 3:**
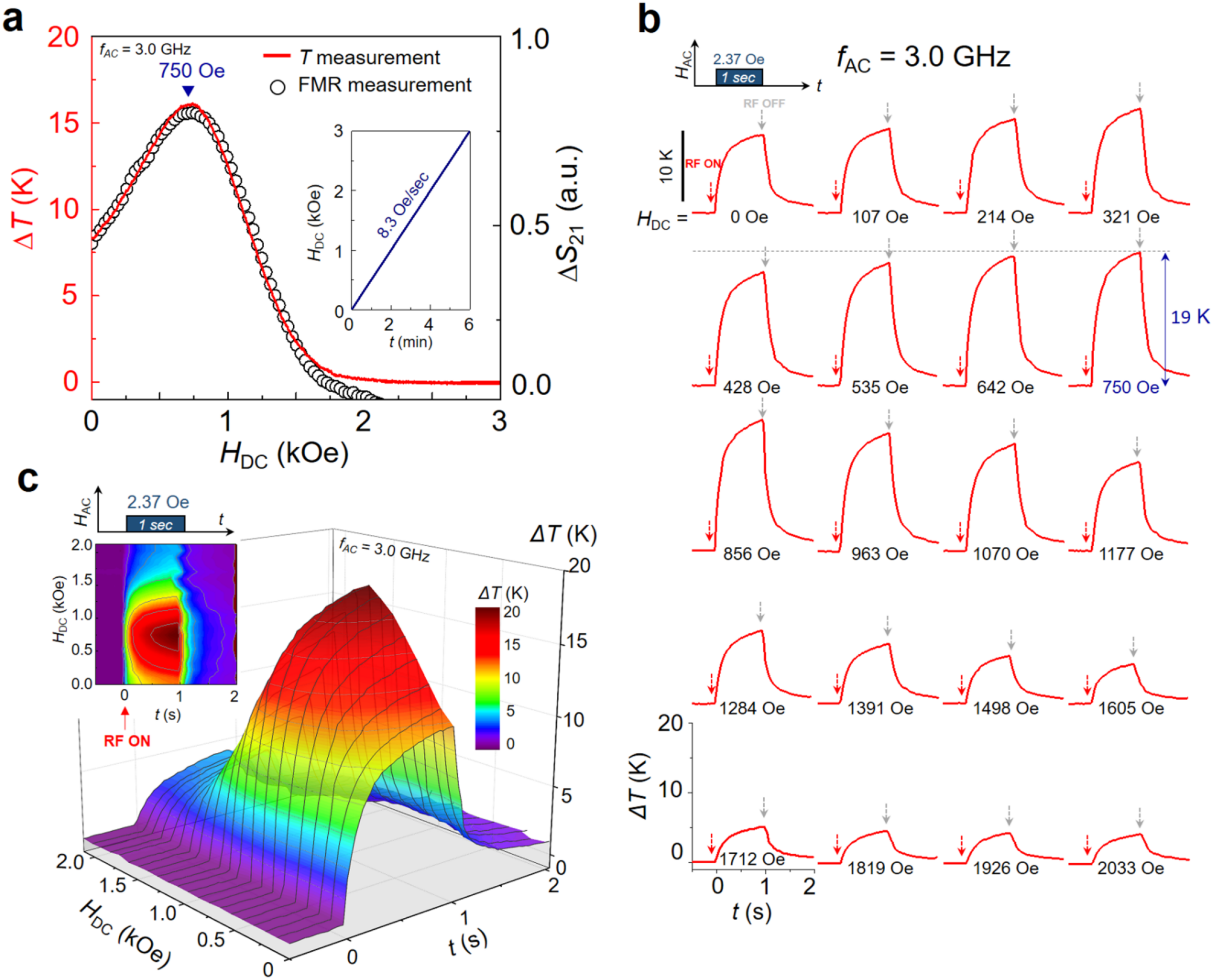
Temperature increments measured under applications of AC and DC magnetic fields. (**a**) Variation of temperature increment ∆*T* (red solid curve) with strength of DC magnetic field that varies proportionally with time at constant rate of 8.3 Oe/sec (as shown in inset) for application of RF magnetic field of frequency *f*_AC_ = 3.0 GHz and strength *H*_AC_ = 2.37 Oe. The open circles represent the microwave absorption spectrum obtained from VNA-FMR measurements under the same resonance condition (for details, see Supplementary Section S4). (**b**) Temperature variation with *t* for different *H*_DC_ values (*H*_DC_ = 0 ~ 2033 Oe with steps of 106 Oe) under application of 1 s-pulse RF magnetic field (*f*_AC_ = 3.0 GHz and *H*_AC_ = 2.37 Oe). (**c**) Corresponding perspective and in-plane (inset) contours on *H*_DC_-*t* plane. The temperature increments are indicted by the color-bar scale.

Figure 3a plots the variation of temperature increment Δ*T* (red solid line) of Fe_3_O_4_ particles with *H*_DC_, which is proportionally increasing with time at a rate of 8.3 Oe/sec (see inset of Fig. 3a), under application of an RF magnetic field at *f*_AC_ = 3.0 GHz and *H*_AC_ = 2.37 Oe (this field frequency corresponds to the resonance pumping at *H*_DC_ = 750 Oe). There are several features from the Δ*T*-*vs*-*H*_DC_ curve: (1) Upon application of the RF field with zero DC field (*H*_DC_ = 0), a sudden temperature increase of Δ*T* = 8 K occurs within a 1 s duration; (2) then, with increasing *H*_DC,_ Δ*T* increases further, monotonically, to its maximum value, Δ*T* = 16 K at *H*_DC_ = 750 Oe; (3) as *H*_DC_ further increases, the particle temperature starts to decrease and converges to the initial equilibrium temperature of 27℃ (Δ*T* ~ 0). The *H*_DC_ = 750 Oe is the DC field strength at which the resonance frequency *f*_R_ of the precession of **M** in the Fe_3_O_4_ is 3.0 GHz. Thus the frequency *f*_AC_ = 3.0 GHz of applied RF field leads to the resonant precession of **M** in the Fe_3_O_4_ particles. This means that the temperature increment is maximized at the resonant excitation. The decrease of Δ*T* with further increase of *H*_DC_ starting from *H*_DC_ = 750 Oe is ascribed to the non-resonance effect of the **M**’s precession motion. The increment of Δ*T* = 8 K even at *H*_DC_ = 0 is owed to the presence of a certain strength of local internal field within the nanoparticles. In our case, the internal field originates mainly from the magnetocrystalline anisotropy, and also in part from inter-particle dipolar interaction due to aggregation of the Fe_3_O_4_ nanoparticles. The particles’ size distribution and surface imperfections through their intra-dipolar interaction are other sources of the internal field. (Further discussion on the internal magnetic field can be found in Supplementary Sects. 3 and 4.) As for the characteristic behaviors of the Δ*T* vs. *H*_DC_ curve, they are exactly correspondent with the measured FMR spectrum of the Fe_3_O_4_ particles (open circles), as shown by the |Δ*S*_21_| vs. *H*_DC_ spectrum (for details on the FMR measurements, see Supplementary Section S3). This agreement was also confirmed by micromagnetic simulations (see Supplementary Section S4), evidencing that the temperature increments are caused purely by heat generation associated with the energy dissipation due to damping against the magnetization precession.

On the basis of the above experimental demonstration of temperature increments from nanoparticles, we further measured the Δ*T* variation as a function of time under the same resonance condition, i.e., the application of the RF field (*f*_AC_ = 3.0 GHz and *H*_AC_ = 2.37 Oe) in a single pulse of 1-s-duration time. Figure 3b illustrates the results of Δ*T* vs. *t* for different *H*_DC_ values along with Fig. 3c’s corresponding perspective view of the *H*_DC_-*t* plane. All of the shapes of Δ*T* vs. *t* have a similar trend, which represents an initially rapid increase followed by a slow further increase and then a sudden drop at RF field-off. The slow increase after the initial fast increase in the particle temperature is due to heat conduction from the particles in direct contact with the sample stage (a hydrocarbon/ceramic substrate). Remarkable differences between the individual *H*_DC_ strengths are the initial temperature increment rate *κ* and, consequently, the maximum temperature increment available from a given DC field strength. For example, under the resonance condition of *H*_DC_ = 750 Oe for *f*_AC_ = 3.0 GHz, we obtained the largest temperature increment of Δ*T* = 19 K for just a 1 s RF-field pulse along with the highest temperature increment, Δ*T* = 10 K, in 0.1 s. Note that the experimentally observed temperature increments were achieved with the extremely-low input power of 5 Watts (*H*_AC_ = 2.37 Oe). The higher the strength of *H*_AC_ with the other parameters remaining constant, the greater the temperature increment (see Fig. S7d). Temperature cooling immediately occurs upon turning off of the RF field, showing classical exponential cooling trends similar to those of the temperature increments. To sum up, the largest peak in Δ*T* on the *H*_DC_-*t* plane shown in Fig. 3c indicates that the resonant RF pulse field allows for a sufficiently fast temperature increment and, consequently, possible targeted heating from the Fe_3_O_4_ nanoparticles through the sustainable spin-excitation and relaxation process.

### Initial temperature increment rate *κ*

On the other hand, in a non-adiabatic system, samples start to lose heat mainly through conduction to the environment when the temperature of the sample is higher than that of the surroundings. Measurement of the initial temperature increment rate d*T*/d*t|*_∆*t*=0_ immediately upon application of the RF field can provide a good linear approximation to an ideal adiabatic system. Thus, heat loss can be assumed to be negligible at the initial stage of fast heat transfer, and *κ* = d*T*/d*t|*_∆*t*=0_ can be expressed by a parameter representative of the heating efficiency of magnetic particles, namely $SLP = \left( {{{C_{{\text{S}}} } \mathord{\left/ {\vphantom {{C_{{\text{S}}} } {m_{{{\text{NP}}}} }}} \right. \kern-\nulldelimiterspace} {m_{{{\text{NP}}}} }}} \right)\left( {{{{\text{d}}T} \mathord{\left/ {\vphantom {{{\text{d}}T} {{\text{d}}t}}} \right. \kern-\nulldelimiterspace} {{\text{d}}t}}} \right)_{t = 0}$, where $C_{{\text{S}}}$ and $m_{{{\text{NP}}}}$ are the specific heat capacity and the mass of magnetic particles, respectively.

**Figure 4:**
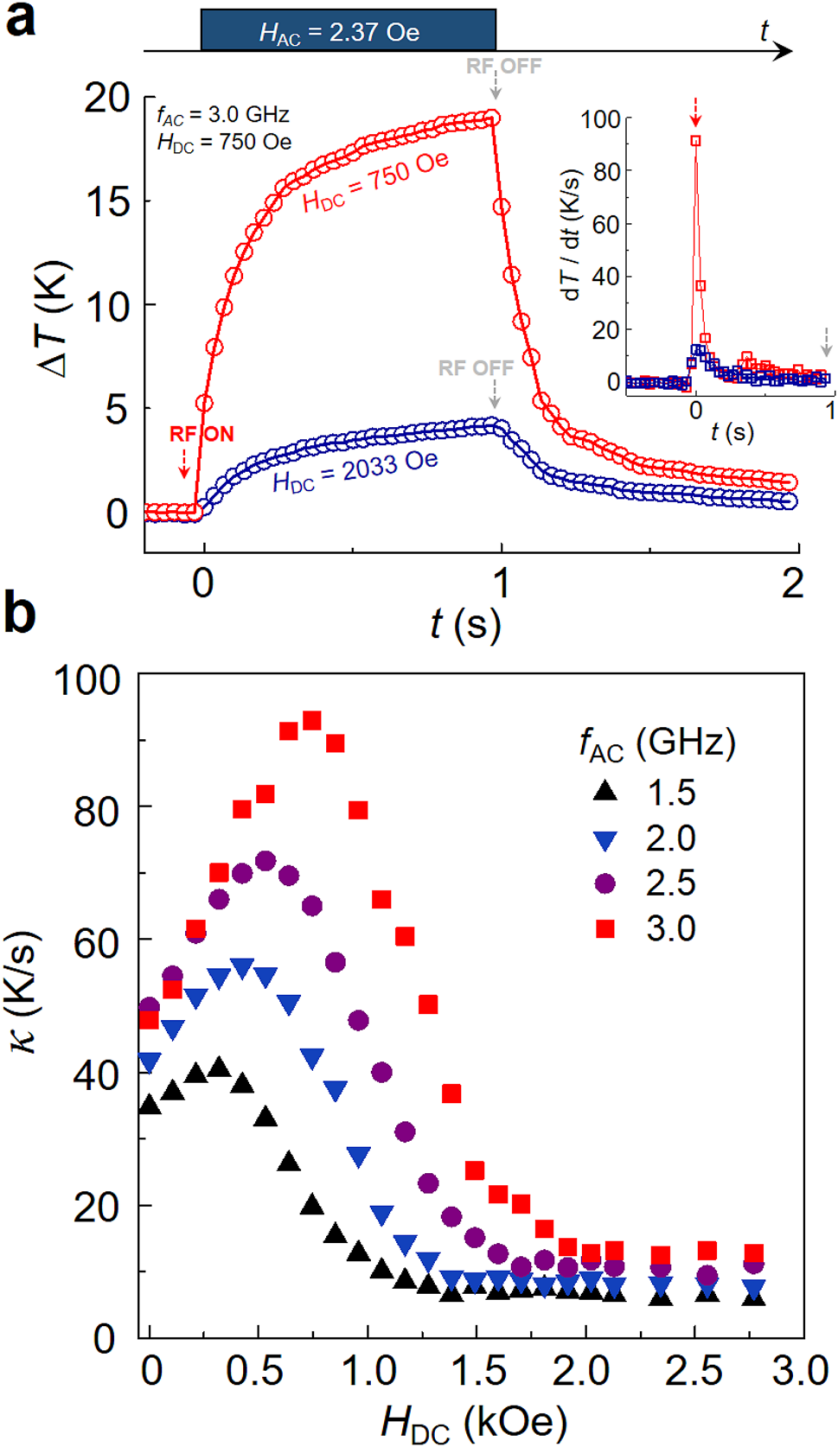
Initial temperature increment rate, *κ.* (**a**) Temperature increments of magnetic nanoparticles measured under application of RF magnetic field of *f*_AC_ = 3.0 GHz and *H*_AC_ = 2.37 Oe for 1 s duration time at both in-resonance (red symbol, *H*_DC_ = 750) and off-resonance (blue symbol, *H*_DC_ = 2033 Oe). The inset shows the time derivatives of the temperature variation, d*T*/d*t*. (**b**) Initial temperature increment rate (*κ* is defined as d*T*/d*t*|_∆t=0_) as function of strength of DC magnetic field for different frequencies, *f*_AC_ = 1.5 (black, up triangles), 2.0 (blue, down triangles), 2.5 (purple circles), and 3.0 GHz (red squares). For all of the measurements, a constant strength of AC field, *H*_AC_ = 2.37 Oe, was applied.

In order to estimate *κ* from our experiments, we plotted Δ*T* vs. *t* for a 1-s RF-field pulse of *f*_AC_ = 3.0 GHz and *H*_AC_ = 2.37 Oe for two different examples, in-resonance (*H*_DC_ = 750 Oe) versus off-resonance (*H*_DC_ = 2033 Oe), as shown in Fig. 4(a). As noted earlier, under the resonance condition (red symbols), the temperature increment was observed to reach 19 K within 1 s. Contrastingly, under the off-resonance condition (blue symbols), the temperature increment was as small as 4 K. This remarkable difference between the resonance and off-resonance cases could be further clarified by the contrasting *κ* values of the time-derivative Δ*T* profiles, as shown in the inset of Fig. 4a. For resonance case, *κ* reached 93 K/s, which value is astonishingly high, especially as compared with *κ* = 12.5 K/s observed for off-resonance case. Most earlier studies have reported that *κ* is much less than ~ 1 K/s for conventional magnetic hyperthermia using a kHz-frequency oscillating field in liquid media^23,24^. To our best knowledge, the highest values of *κ* ~ 3 K/s have been obtained for dried powder states of NiFe_2_O_4_^25^, MgFe_2_O_4_^26^, or Mg-doped γ-Fe_2_O_3_^13^. Table 1 shows the comparison of heating powers SLP and *κ* obtained under different experimental conditions of AC magnetic fields for a variety of magnetic nanoparticles, as found in the literature. Our observation of such an extremely high value as *κ* = 93 K/s evidences that the resonant spin-excitation and relaxation mechanism is very promising for establishment of fast local heating for highly efficient hyperthermia treatment with extremely low-power RF magnetic field (e.g., *H*_AC_ < 5 Oe) and DC magnetic field strengths (*H*_DC_ < 1 kOe).

**Table 1:** Comparison of heating powers (SLP and *κ*) reported in literature.

Type of materials	Size (nm)	AC field parameters		Heating parameters			References
		*f* (kHz)	*H*_AC_ (Oe)	SLP (W/g)	Environment	*κ*^a)^ (K/s)	
Fe_3_O_4_	24	260	160	137	Liquid Medium	0.16	^27^
MnFe_2_O_4_@CoFe_2_O_4_	15	500	469	3034		~ 1.5^b)^	^11^
α-Fe_2_O_3_ nanoring	70	400	440	2213		0.17	^28^
faceted ferrite	19	250	251	582		0.28	^29^
Mg-doped γ-Fe_2_O_3_	7	110	140	–	Dried Powder State	~ 3	^13^
NiFe_2_O_4_	54	10	500			2.5	^25^
MgFe_2_O_4_	101	110	140			2.2	^26^
commercial Fe_3_O_4_	50	313	340			2.3	^30^
Fe_3_O_4_	15	3 (GHz)	3			150	This work

a) The values of *κ* were obtained by measuring the initial slopes of temperature*-vs*-time curves found in the literature, except for the value of b).

b) This value was estimated by a numerical conversion from the SLP value reported in the corresponding reference.

For comparison with other frequencies, we plotted *κ* versus *H*_DC_ for *f*_AC_ = 1.5, 2.0, 2.5 and 3.0 GHz, as shown in Fig. 4b (see Supplementary Fig. S7a–c for all of the Δ*T* − *t* plots). All of the *κ* values as a function of *H*_DC_ exhibited a similar trend but different maximum values of *κ* = 40, 56, 72, 93 K/s at their corresponding DC fields for resonance, *H*_DC_ = 321, 428, 535, and 750 Oe for *f*_AC_ = 1.5, 2.0, 2.5, and 3.0 GHz, respectively. Those peak positions corresponded to the resonance static fields of the given frequency of RF field as previously noted (note that the intrinsic precession frequency, *f*_R_ = 1.5, 2.0, 2.5, and 3.0, is given for *H*_DC_ = 321, 428, 535, and 750 Oe, respectively).

Another feature we observed is that the *κ* values in the higher *H*_DC_ range converged to a specific low value, about 10 K/s or less. This non-zero, small value of *κ* even under the off-resonance condition, could be ascribed to a dielectric origin of power loss in magnetic nanoparticles, as evidenced by the further electromagnetic numerical calculations shown in Supplementary Section S5. The results shown in Fig. 4b indicate that very high temperature increment rates can be achievable, and reliably controllable, only by adjusting the parameters of the externally applied RF fields as well as the DC field strength.

### Delicate control of local heating by magnetic field parameters

**Figure 5:**
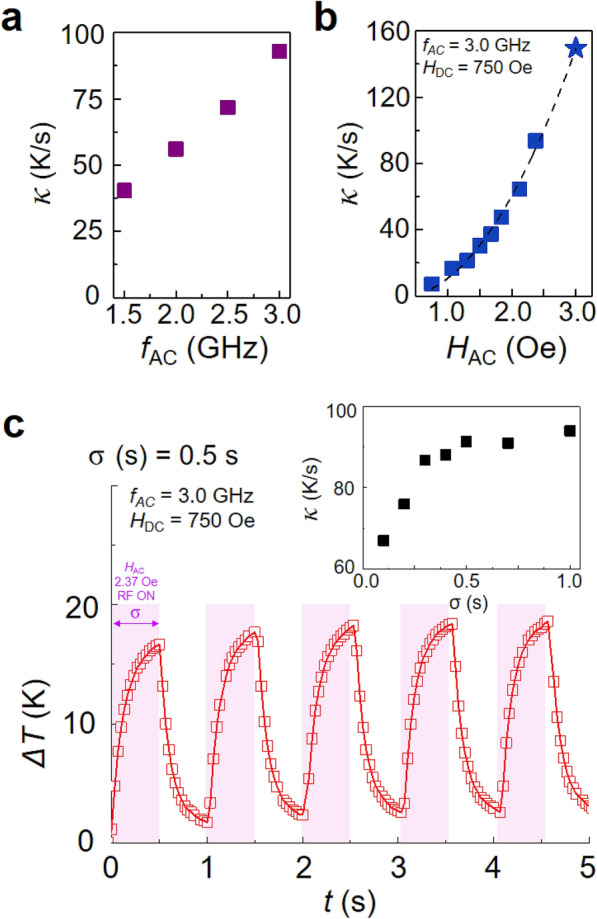
External field parameter dependence of initial temperature increment rate *κ*. (**a**) Maximum *κ* values versus *f*_AC_ obtained from Fig. 4b. (**b**) *κ* values versus *H*_AC_ at resonance under application of 1-s-duration pulse of RF field at *f*_AC_ = 3.0 GHz with *H*_DC_ = 750 Oe. The blue star symbol corresponds to *κ* = 150 K/s found at *H*_AC_ = 3 Oe, which was the highest value observed in our experiments. The dashed curve indicates a quadratic fit ($\kappa = 15.82\,H_{{{\text{AC}}}}^{2}$) to the experimental data. (**c**) Temperature increments for periodic pulses (pulse width σ = 0.5 s) of resonant RF magnetic field of *f*_AC_ = 3.0 GHz and *H*_DC_ = 750 Oe at *H*_AC_ = 2.37 Oe. The inset shows the *κ* variation with the pulse width σ.

In addition to the controllable amount of heat generated from the Fe_3_O_4_ particles by the strength of DC magnetic fields, we also measured the maximum values of *κ* for different values of *f*_AC_ and *H*_AC_. The maximum *κ* values for the indicated frequencies were estimated from Fig. 4b, which reveals a linear proportion to *f*_AC_, as plotted in Fig. 5a. On the other hand, the *κ* value as a function of *H*_AC_ showed a quadratic dependence under the resonance condition (*f*_AC_ = 3.0 GHz and *H*_DC_ = 750 Oe), as indicated by the fitting curve ($\kappa = 15.82H_{{{\text{AC}}}}^{2}$) to the experimental data (see Supplementary Fig. S7d for Δ*T*-*t* plots with different *H*_AC_ strength). The highest value among our observation data was *κ* = 150 K/s (see the blue star symbol) at *H*_AC_ = 3 Oe under the resonance condition of *f*_AC_ = 3.0 GHz and *H*_DC_ = 750 Oe. Although the *H*_AC_ = 3.0 Oe value was limited in our experimental setup, the fitting curve indicates that *κ* can be increased further with *H*_AC_. The observed value of *κ* = 150 K/s is, to our best knowledge, significantly higher than any of the experimentally observed values recorded thus far for all other magnetic heat induction techniques^13,23–30^.

Next, we further examined *κ* under application of several numbers of pulses (pulse width σ = 0.1–1.0 s) of a resonant RF field (again, *f*_AC_ = 3.0 GHz, *H*_DC_ = 750 Oe with *H*_AC_ = 2.37 Oe), as shown in Fig. 5c. The temperature was increased and then dropped, repeatedly, according to the pulse being on and then off. The available *κ* values with different pulse widths are plotted in the inset. For σ > 0.3 s, *κ* was maximized to 90 K/s, while *κ* was reduced with decreasing σ for σ < 0.3 and turned out to be *κ* = 67 K/s for σ = 0.1 s. For smaller values of σ less than 0.1 s, we could not measure *κ*, due to the limited temporal resolution (30 Hz) of the IR camera used in our measurement (for the Δ*T*-*t* curve for σ = 0.05 s, see Supplementary Fig. S7e). The results in Fig. 5 reveal that the parameters of the externally applied RF fields, such as *H*_AC_, *f*_AC_, σ as well as *H*_DC_, allow for reliably delicate manipulation of *κ*, thereby enabling well-controllable local heating (temperature increment) around the Fe_3_O_4_ particles. Furthermore, the delicate controls of these field parameters are readily available, and also, the strengths of the RF and DC magnetic fields used were relatively very low, as weak as a few Oe for the RF field and a few kOe for the DC field.

## Discussion

In summary, we experimentally demonstrated a novel concept of high-efficiency heat generation based on resonant spin-excitation and dissipation, which has never been demonstrated in the context of magnetic hyperthermia. In particular, we observed an unprecedented value of initial temperature increment rate (*κ* ~ 150 K/s), even with very low power consumption, as small as a few Oe of RF magnetic field. The extremely high value of *κ* allows for local heating as fast as 0.1 s. Furthermore, this mechanism enables a delicate control of local heating by adjustment of several field parameters, i.e., the strengths of DC/AC magnetic fields, the frequency of the AC field, and its pulse width. Our approach can provide key insights into a new direction toward successful magnetic heat-treatment solutions to overcome the current limitations of conventional magnetic hyperthermia. Its rapid temperature increment would allow a remarkably fast response^31^; and the dosage of magnetic nanoparticles required for sufficient heat generation/dissipation, therefore, can be much reduced. On the other hand, the frequency of AC magnetic fields for this new technique is higher than the Brezovich limit (*f*∙*H* = 4.85 × 10^8^ A/m∙s)^32^, which is the maximum of the product of AC magnetic field strength and frequency required for human safety from high–frequency field exposure. However, clinically available MRI using RF magnetic field strengths of 10–100 μT and frequencies up to 500 MHz (at 11.7 T MRI)^33^ give rise to *f*∙*H* = 4 × 10^10^ A/m∙s, which far exceeds the Brezovich’s acceptable limit. Our proposed technique has *f*∙*H* < 7 × 10^11^ A/m∙s, which would be clinically acceptable by using short pulse field exposure and also AC field frequency of less than GHz with larger particle sizes^18^. For successful clinical implementations in the near future, however, it is necessary to conduct *in-vitro* and *in-vivo* experimental demonstrations based on resonant spin-excitation and consequent dissipation in magnetic nanoparticles injected into tumors. In the meantime, this work offers a first step towards a paradigmatic shift in magnetic hyperthermia that would make possible successful medical treatment based on utilization of FDA-approved magnetic nanoparticles.

## Methods

### Measurements of temperature increment from Fe_3_O_4_ particles

Using a drop-casting method, the Fe_3_O_4_ nanocrystals (about 1 mg) were placed on the surface of a 400-μm-length Cu line in a microstrip consisting of a central waveguide on the top layer, a ground plane on the bottom layer, and a low-loss hydrocarbon/ceramic substrate (RO4003) between them. To allow spin excitation in the particles, AC currents of different GHz frequencies using a signal generator (E8257D, Agilent) were applied to the microstrip on which the nanoparticles had been placed, and were amplified to several watts by an RF power amplifier (5170FT, Ophir) to generate sufficient strengths of AC magnetic fields around the signal line. The experimental setup also includes an electromagnet for application of DC magnetic fields along the microstrip line. The AC magnetic fields were applied in the direction perpendicular to the DC field direction. An infrared (IR) camera (T650sc, FLIR) was used to measure real-time temperature increments in the samples from thermal radiation at an accuracy of about ± 1 K and a maximum temporal resolution of 30 Hz. The numerical values of temperature were determined by averaging local temperatures in an area of 200 μm × 200 μm at the center of the signal line. In order to read the exact temperature of the magnetic nanoparticles, the parameter values for the calculation are as follows: infrared emissivity of Fe_3_O_4_ nanoparticles^34^ $\varepsilon_{{{\text{Fe}}_{{3}} {\text{O}}_{{4}} }} = 0.97$ (very close to emissivity of black body, $\varepsilon = 1$), temperature of atmosphere *T*_atm_ = 25 °C, distance from IR camera to sample *D* = 50 cm. We also measured the temperatures of ice and boiling water in order to confirm the calibration and accuracy (see Supplementary Section S2).

## Supplementary Information

Supplementary Information.
