# Supplementary material for: Ultra-high rate of temperature increment from superparamagnetic nanoparticles for highly efficient hyperthermia
Source: Sci Rep. 2021 Mar 2;11:4969. doi: 10.1038/s41598-021-84424-1 (PMC7925677; doi:10.1038/s41598-021-84424-1)
Supplement: Supplementary file 1 — Supplementary Information. [file 41598_2021_84424_MOESM1_ESM.pdf]

Ultra-high rate of temperature increment from superparamagnetic nanoparticles for highly efficient hyperthermia

Jae-Hyeok Lee[1], Bosung Kim[1], Yongsub Kim[1], and Sang-Koog Kim[1,*]

[1] *National Creative Research Initiative Center for Spin Dynamics and Spin-Wave Devices, Nanospinics Laboratory, Research Institute of Advanced Materials, Department of Materials Science and Engineering, Seoul National University, Seoul 151-744, South Korea*

Supplementary Section 1 | Synthesis of Fe_3O_4 nanoparticles and their structural and magnetic characteristics

Supplementary Section 2 | Calibration of temperatures measured using IR camera

Supplementary Section 3 | Magnetization dynamics of Fe_3O_4 particles

Supplementary Section 4 | Micromagnetic numerical calculation of dynamics of single spherical Fe_3O_4 particle of 15 nm diameter

Supplementary Section 5 | Electromagnetic calculation of temperature increment of particles by dielectric loss

Supplementary Section 6 | Curves of temperature increment *vs* time according to different external field parameters

[a)] Correspondence and requests for materials should be addressed to S.-K.K. (sangkoog@snu.ac.kr).

S1. Synthesis of Fe_3O_4 nanoparticles and their structural and magnetic characteristics

A. Synthesis: Fe_3O_4 nanoparticles were synthesized through thermal decomposition of $Fe(acac)_3$ (acac=acetylacetonate) in a hot organic solvent, using a previously developed and reported approach.[1] $FeCl_2$ (0.420 g) and $Fe(acac)_3$ (1.765 g), oleylamine (20 ml), oleic acid (5 ml), and trioctylamine (15 ml) were dissolved under an argon atmosphere. The resulting mixture was heated to 300°C and refluxed for an hour. After cooling to room temperature, black-colored magnetite nanocrystals were isolated by adding an excess amount of ethanol and subsequent centrifugation. In order to make silica-coated shell around the magnetic nanoparticles, the mixture solution containing the Fe_3O_4 nanoparticles (1 mg), Igepal CO-520 (800 mg), ammonium hydroxide (105 μl), cyclohexane (12 ml) and tetraethyl orthosilicate (40 μl for the silica shell of 15 nm) was maintained at room temperature for 72 h. $Fe_3O_4@SiO_2$ nanoparticles were collected by centrifugation after the addition of an excess amount of ethanol, followed by purification using a MACS column (Miltenyi Biotec) and dispersion in water after carboxylate functionalization.

B. Structural and magnetic properties: The diameter and shape of the Fe_3O_4 nanoparticles were examined by measurement of bright-field transmission electron microscopy (TEM) images. Figure S1(a) shows an example a TEM image, representing highly monodispersed and spherical particles of an average diameter of d = 15 nm, as well as the separation of the individual nanoparticles in silica shells (Fig. S1(b)). The utility of the silica-shell coating around the core particles is the suppression of magnetic interactions between nanocrystals. The mean value of the sizes of nanocrystals without silica shells was estimated to be 15.3 (± 0.74) nm, as shown in the histogram of the particle size distribution (Fig. S1(c)). The field-decreasing

magnetization curve (Fig. S1(d)) of the Fe_3O_4 nanoparticles prior to silica coating exhibits the characteristics of superparamagnets with zero coercivity and zero remanence at 300 K (saturation magnetization M_S = 102.24 emu/g, similarly to a previous report[2]).

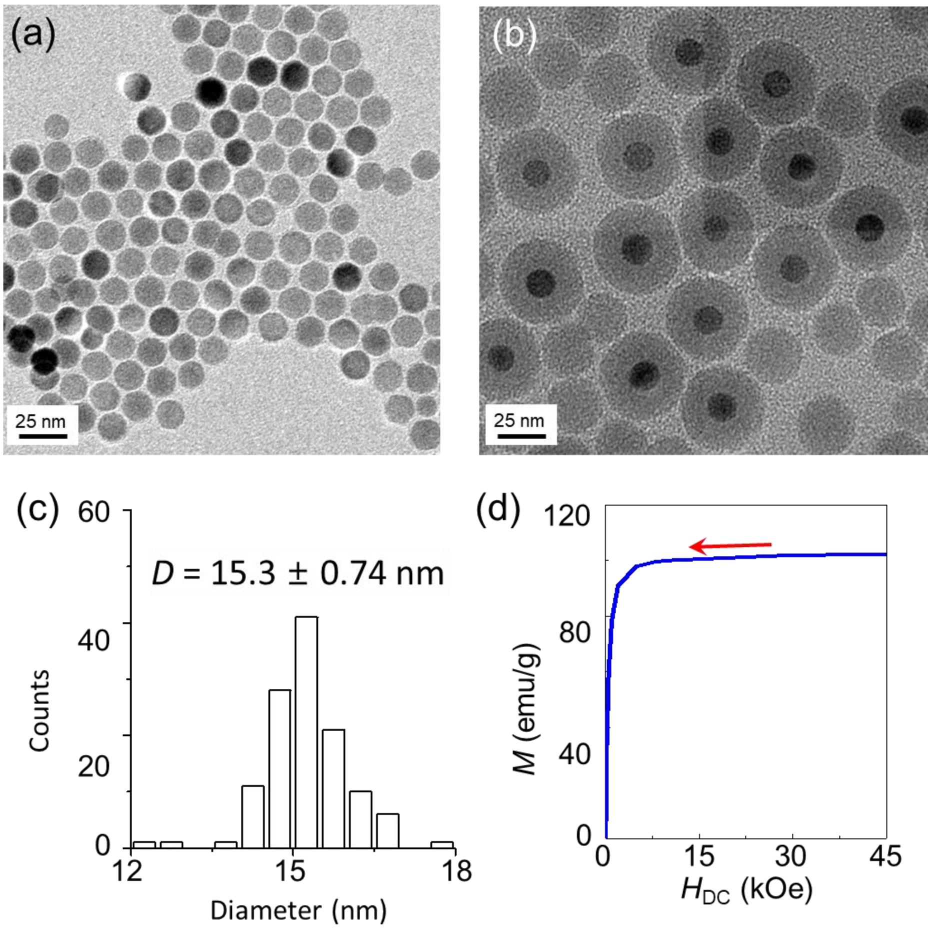

Figure S1. TEM images of (a) Fe_3O_4 nanocrystals and (b) Fe_3O_4 capped with silica shells. (c) Histogram of size distribution of Fe_3O_4 nanocrystals without silica shells, with mean value of 15.3 nm and standard deviation of 0.74 nm. (d) Room-temperature magnetization curve for Fe_3O_4 nanocrystals without silica shells, as measured by VSM under decreasing magnetic field from 45 kOe to zero.

S2. Calibration of temperatures measured using IR camera

The infrared (IR) thermography employed in this study detects thermal radiation phenomena based on the black-body radiation theory. For non-black body (or gray-body) radiator, this is done according to the Stefan-Boltzmann formula. The intensity of radiation emitted by the sample is given as $W = \varepsilon\sigma T^4$ [W/m^2], with ε the infrared emissivity of the sample ($\varepsilon < 1$) and σ the Stefan-Boltzmann constant ($\sigma = 5.67\times10^{-8}\ \mathrm{W\cdot m^{-2}\cdot K^{-4}}$). The IR camera used in our experiment to record the temperature of magnetic nanoparticles receives radiation not only from the sample itself but also from the surroundings reflected via the sample surface. Both of these radiation contributions become attenuated to some extent by the atmosphere between the sample and the IR camera. Here, we briefly present the general formula for accurate measurement of sample temperature and recording by IR camera. The voltage signal of the radiation emitted by sample V_s, which is recorded by IR camera, is proportional to the radiation intensity W from a sample of temperature T_s and can be expressed as[3] $V_\mathrm{s} = C\cdot W(T_s)$, with C a proportional constant. Assuming that the reflected temperature T_refl is the same for all emitting surfaces and that the emittance for the surroundings is equal to unity, the total received radiation intensity can be written $W_\mathrm{total} = \varepsilon\tau W_\mathrm{s} + (1-\varepsilon)\tau W_\mathrm{refl} + (1-\tau)W_\mathrm{atm}$, with τ the spectral transmittance of the sample. Therefore, the voltage signal only from the sample can be summarized as $V_\mathrm{s} = \frac{1}{\varepsilon\tau}V_\mathrm{tot} - \frac{1-\varepsilon}{\varepsilon}V_\mathrm{refl} - \frac{1-\tau}{\varepsilon\tau}V_\mathrm{atm}$.

To ensure the reliability of temperatures measured by the IR camera, we confirmed the temperatures of melting ice and boiling water, which were measured to be −1.2°C and 99.2°C, respectively (see Fig. S2). In the measurement, IR emissivity ε was chosen to be 0.97 for the ice and 0.98 for the boiling water, according to Ref. 4. Note that the measurement accuracy

guaranteed by the manufacturer of the IR camera, noted above, is about ±1 K, which agreed with our measured values. Therefore, IR thermography can be considered to be a reliable non-contact method for measurement of temperature to an accuracy of about ±1 K.

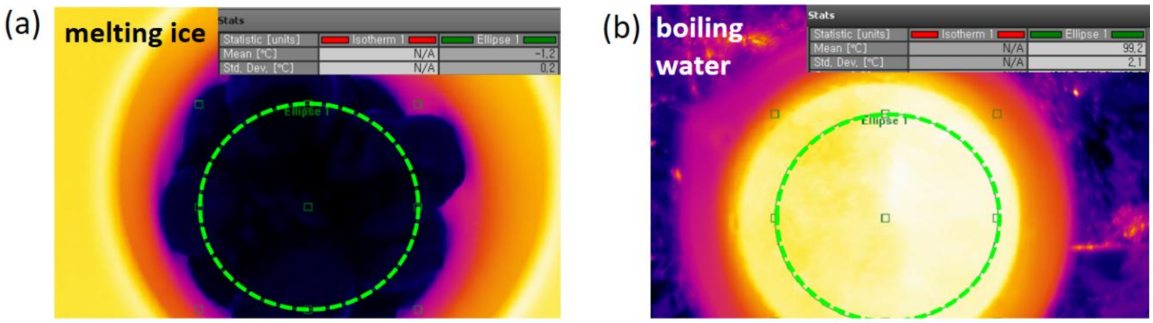

Figure S2. IR images of distribution of local temperatures in (a) melting ice and (b) boiling water. The insets represent the mean and standard deviation values for the area inside each green-dotted circle.

S3. Magnetization dynamics of Fe_3O_4 particles

A. Measurements

To measure FMR, a high-frequency RF oscillating magnetic field h_{RF} at the FMR frequency is necessary to drive the resonance in magnetic materials. This RF magnetic field can be generated around a waveguide line when the RF current I_{RF} from an RF signal-generating source (such as VNA) transmits through a microstrip. When a magnetic sample is placed on the microstrip, resonance can be induced by absorbing the energy from the RF magnetic field. The resonant condition can be expressed as non-zero time-averaged absorbed power[5] $P_{tot} = \left\langle h_{RF} \frac{d\mathbf{m}}{dt} \right\rangle_t \neq 0$. In order to examine the magnetization-dynamic behavior of the Fe_3O_4 particles, we used a vector network analyzer-ferromagnetic resonance (VNA-FMR) approach,[6] as schematically shown in Fig. S3. The sample was prepared in the same manner as described in Method. The microstrip was connected to a VNA (E8362C, Agilent) and placed between the poles of an electromagnet so that DC magnetic fields could be applied parallel to the longitudinal direction of the waveguide. The coaxial cables for high-frequency measurements were equipped with Teflon insulation of 18 GHz bandwidth. Non-magnetic End Launch SMA-type connectors to link the microstrip and the cables were used to prevent interference with the applied DC magnetic field. With this setup, we measured the scattering parameter $|S_{21}|$ of the particle samples, where $|S_{21}|$ represents the power transferred from Port 1 to Port 2. Prior to the VNA-FMR measurements, a standard short-open-load-through (SOLT) 2-port calibration procedure was performed to remove systematic errors originating from outside the microstrip device, such as from cables, connectors, and/or the instrument itself. The measurement conditions were as follows: VNA frequency sweep, 50 MHz - 10 GHz; intermediate frequency,

500 Hz; stimulus input power, 0 dBm. The strength of applied DC magnetic fields was increased from 0 to 3 kOe in stepwise increments of 15 Oe every 45 sec. The frequency-sweeping spectrum for the given DC field strengths was obtained by averaging three separate measurements. The measured $|S_{21}|$ spectra were calibrated by subtracting background signals measured at a sufficiently strong magnetic field of H_{DC} = 3.5 kOe corresponding to non-resonant excitation.

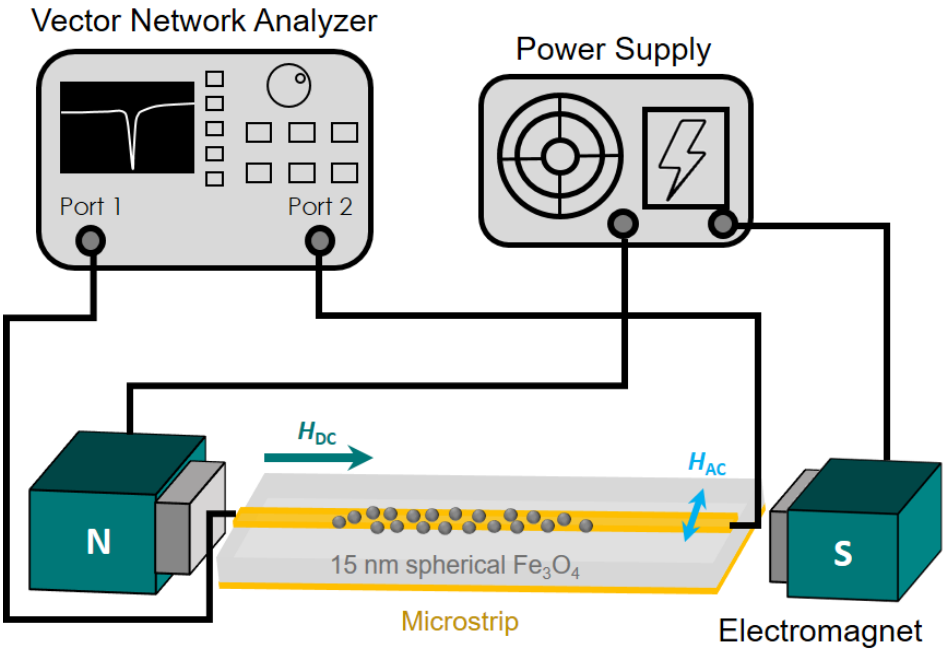

Figure S3. Schematic drawing of VNA-FMR set-up along with microstrip on which Fe_3O_4 nanoparticles were placed. The directions of the applied DC and AC magnetic fields are indicated by the respective arrows.

B. FMR spectra

The $|\Delta S_{21}|$ ($=\left|S_{21,H} - S_{21,H=3.5\mathrm{kOe}}\right|$) spectra measured are shown in Fig. S4. The $|\Delta S_{21}|$ contour plots on the f-H_{DC} plane clearly reveal typical FMR behavior of superparamagnetic nanoparticles: the precession motion of the individual spins are resonantly excited at the

corresponding resonant frequency of AC magnetic fields under a given DC field, which strength H_{DC} is sufficiently strong to align most of the magnetizations in the DC field direction (see the spectra in the higher field range). We performed linear fitting to the experimental data only in the region of H_{DC} > 500 Oe, because the magnetization of the nanoparticles above H_{DC} ~ 500 Oe was sufficiently saturated.

C. Estimation of resonance frequency and damping constant

For non-negligible damping, the resonance frequency of precession in a sphere is expressed as[7-9] $f_R = \frac{\gamma_0}{1+\alpha^2}(H_{DC} + H_{int})$, with γ_0 the electron gyromagnetic ratio. Using this equation, a linear fit to the experimental data for silica-shell-coated Fe_3O_4 nanoparticles (Fig. S4(a)) yielded $f_R = (2.64\,\mathrm{MHz/Oe}) \times H_{DC} + 468\,\mathrm{MHz}$, α = 0.246, and H_{int} = 177 Oe for the intrinsic value of γ_0 = 2.8 MHz/Oe. The internal field value of 177 Oe originates predominantly from the magnetocrystalline anisotropy of individual particles with randomly oriented anisotropy axes as well as the intra-dipolar interaction inside each particle, due to the imperfection of the sphere shape and possible disorders on the particle surfaces. The estimated value of α was within the range of the previously reported values, 0.2 - 0.5.[10-12] For comparison, the FMR spectrum of the Fe_3O_4 nanoparticles without silica shell is shown in Fig. S4(b). A linear fit to the spectrum resulted in $f_R = (2.63\,\mathrm{MHz/Oe}) \times H_{DC} + 1018\,\mathrm{MHz}$, from which we obtained α = 0.254 and H_{int} = 387 Oe. This larger internal field relative to the value of 177 Oe obtained for silica-shell-coated Fe_3O_4 particles is ascribable to an intra-dipolar interaction between the neighboring particles in their agglomerated form. The broader linewidth of the FMR spectrum for the Fe_3O_4 nanoparticles without silica shell and the higher frequency shift at zero-field are also attributed

to this stronger inter-particle dipolar interaction and non-uniform distribution of local demagnetization fields in magnetically interacting particles.[13,14]

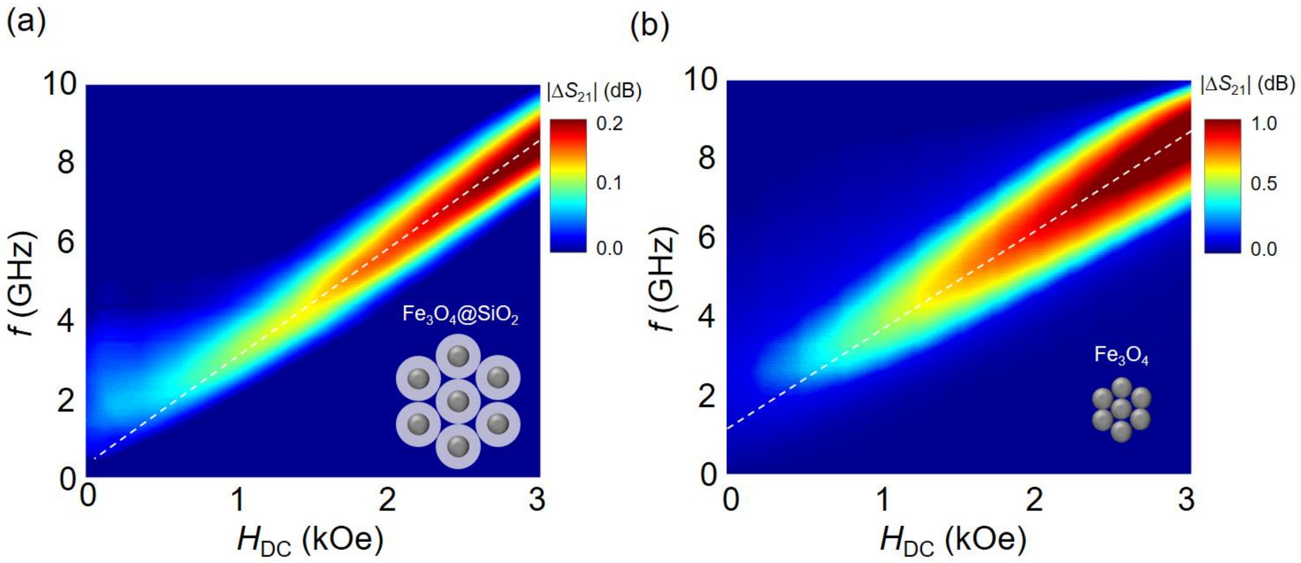

Figure S4. Comparison of spectra of $|\Delta S_{21}|$ on f-H_{DC} plane for (a) silica-shell-coated Fe_3O_4 nanoparticles and (b) nanoparticles without silica-shell. The dotted lines represent linear fits to the experimental spectra using $f_R = \left(\gamma_0 / (1+\alpha^2)\right)(H_{DC} + H_{int})$.

S4. Micromagnetic numerical calculation of dynamics of single spherical Fe_3O_4 particle of 15 nm diameter

The magnetization dynamics of a single spherical Fe_3O_4 particle of $2R$ = 15 nm diameter (for the model geometry, see Fig.S5) were numerically solved using the FEMME code (version 5.0.9).[15] The curved surface of the finite-element model sphere was discretized into triangles of approximately equal area using Hierarchical Triangular Mesh[16] (see Fig. S5(a)). The material parameters for Fe_3O_4 were as follows: saturation magnetization M_S =102.24 emu/g, exchange stiffness A_{ex} = 13.2 pJ/m, and cubic anisotropy constant $K_1 = -1.36 \times 10^4$ J/m^3 with a damping parameter of α = 0.246. The values of A_{ex} and K_1 were obtained from the experimental data reported in Ref. 17, whereas the values of M_S and α were directly measured by VSM and VNA-FMR, respectively. For the simulation, we had assumed randomly distributed magnetic anisotropy directions, and so we used the mean orientation value $\langle \cos\theta \rangle K_1 = 0.5K_1$.[18] For H_{DC} > 500 Oe, the Zeeman energy sufficiently overcomes the thermal energy of spin relaxation in the particles at room temperature. Therefore, our numerical simulation carried out at T = 0 K can represent the general features of the dynamic motions of nanoparticles. In order to determine the resonance frequency of the precession in a single spherical Fe_3O_4 nanoparticle, first we applied a DC field of 1 kOe in the +z-direction (see Fig. S5(b)). After setting the orientations of the initial magnetizations in the direction of the DC field, H_{DC} = 1 kOe, a sinc-function field of $H_{sinc} = H_0 \sin[2\pi f_{sinc}(t-t_0)]/[2\pi f_{sinc}(t-t_0)]$, where H_0 = 5 Oe, was applied along the x-axis for t = 100 ns. Figure S5(c) shows the resultant power spectrum in the frequency domain as obtained from the FFTs of the temporal oscillations of the volume-averaged x-component of the magnetizations. From the spectrum, the resonance frequency of a single Fe_3O_4 magnetic nanoparticle was determined to be f = 3.0 GHz at H_{DC} = 1 kOe, which agreed

well with the value of f_R = 3.0 GHz obtained from the experimental VNA-FMR measurement for the silica-shell-coated Fe_3O_4 nanoparticles (red circles). Also, numerical estimation of an ensemble average over randomly distributed isolated Fe_3O_4 nanoparticles results in the internal field H_{int} = 174, which value is in good agreement with H_{int} = 177 Oe estimated from the experimental FMR measurement for the Fe_3O_4 nanoparticles with silica shells. Figure S5(d) compares the temporal oscillations of the x-component magnetization, m_x=M_x/M_S, excited by f_{AC} = 3 GHz and H_{AC} = 5 Oe, at H_{DC} = 1 kOe (resonance) and H_{DC} = 2 kOe (non-resonance). The much higher oscillation amplitude at H_{DC} = 1 kOe than that at H_{DC} = 2 kOe revealed the resonance excitation by the very low (H_{AC} = 5 Oe) field strength.

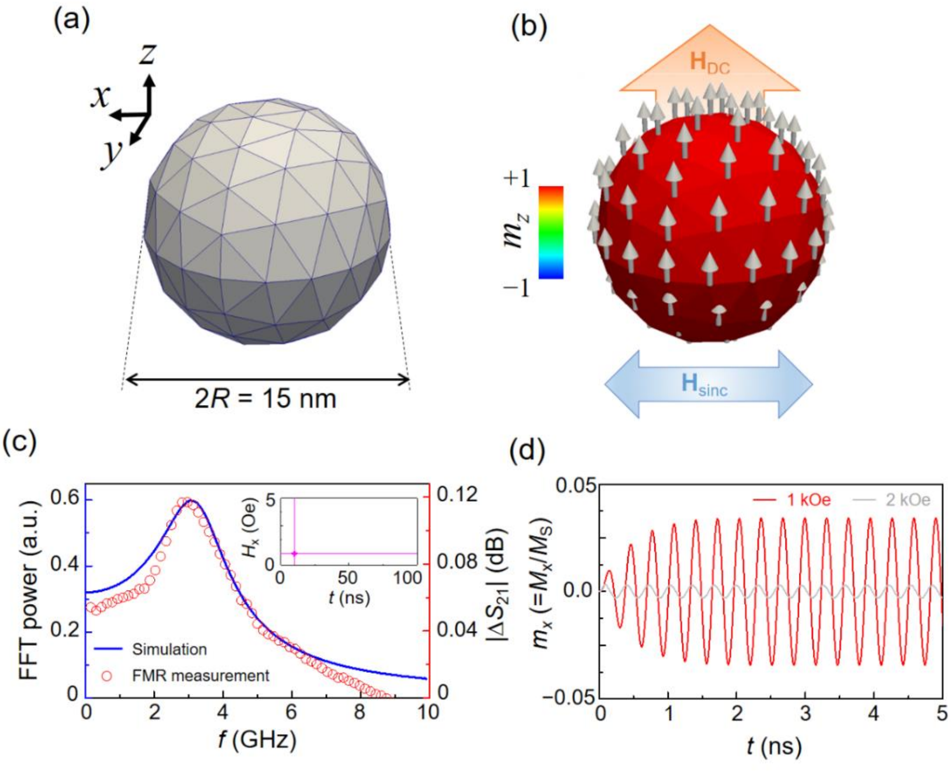

Figure S5. (a) Finite-element meshes of nanosphere of diameter 2R = 15 nm. Mesh sizes are below 2.5 nm (b) Ground-state magnetization configuration in Fe_3O_4 nanoparticle under H_{DC} = 1 kOe. The directions of the applied H_{DC} and H_{sinc} are indicated by the vertical and horizontal arrows, respectively. (c) Frequency spectra as obtained from FFT of m_x oscillation excited by sinc-field at H_{DC} = 1 kOe (blue line) and from measured $|\Delta S_{21}|$ by VNA-FMR experimentation at H_{DC} = 1 kOe. The inset represents the sinc-field applied along the x-axis where $H_{sinc} = H_0 \sin[2\pi f_{sinc}(t-t_0)]/[2\pi f_{sinc}(t-t_0)]$ with H_0= 5 Oe, f_{sinc} = 10 GHz, and t_0 = 10 ns for a period of t = 100 ns. (d) Temporal oscillations of m_x at H_{DC} = 1 kOe (red) and H_{DC} = 2 kOe (gray), along with H_{AC} = 5 Oe and f_{AC}= 3 GHz

S5. Electromagnetic calculation of temperature increment of particles by dielectric loss

In order to understand the experimentally observed temperature increment in the higher DC-field region (off-resonance) as shown in Fig. 4(b), we numerically calculated the power dissipation caused by dielectric loss (P_{dielect}) when exposed to a microwave field. P_{dielect} is given as[19] $P_{\text{dielect}}(\text{W}/\text{m}^3) = \pi f \varepsilon_0 \varepsilon_r'' |E|^2$ with ε_0 the vacuum permittivity and ε_r'' the imaginary part of the complex relative permittivity of a given material, and *E* the electric field applied to the sample. To obtain the spatial distribution of the electric field amplitude |*E*| in a 50 μm-thick strip of Fe_3O_4 particles, we conducted an electromagnetic simulation using the CST microwave studio for a given model geometry, as shown in Fig. S6(a). The spatial distributions of both the electric and magnetic fields (*E* and *H*) for f_{AC} = 3.0 GHz with an input power of 1 W were calculated as shown in Fig. S6(b). The volume-averaged values of |*E*| and |*H*| over the 50 μm-thick Fe_3O_4 strip were estimated to be 11,561 V/m and 84.4 A/m (= 1.06 Oe), respectively. Assuming that the power dissipated in the particle strip is completely converted into heat under adiabatic conditions, the temperature increment rate d*T*/d*t* was given as $\text{d}T/\text{d}t = P_{\text{dielect}} / C_{Fe_3O_4} = \pi f \varepsilon_0 \varepsilon_r'' |E|^2 / C_{Fe_3O_4}$ with $C_{Fe_3O_4}$ the volumetric specific heat capacity of Fe_3O_4 ($C_{\text{Fe}_3\text{O}_4} = 3.367 \times 10^6\ \text{J/K} \cdot \text{m}^3$). Using the material parameters for Cu, RO4003, and Fe_3O_4 shown in Table S1, the power density from the dielectric loss in the Fe_3O_4 strip for f_{AC} = 3.0 GHz was calculated as $P_{\text{dielect}} = 5.35 \times 10^6\ \text{W/m}^3$ for an input power of 5 W. The temperature increment rate was then estimated to be $dT/dt = 7.95\,\text{K/s}$. Also, for the other frequencies, we calculated d*T*/d*t* using |*E*| distributions obtained from the simulation and then compared them with the experimental value obtained at H_{DC} = 2.5 kOe (off-resonance condition), as shown in Fig. S6(c). The experimental and simulation values showed the same trend, that of a linear increment in d*T*/d*t* with the AC field frequency, but the experimental

values (black squares) of dT/dt were, in general, higher by 1-5 K/s than the simulation values (red squares), The discrepancy between the experimental and simulation data was likely due to air-gaps between the Fe_3O_4 nanoparticles in the real samples, which had not been assumed in the model simulation. Another factor could have been the Joule's heating arising from the conductive Cu line and/or the Fe_3O_4 particles in the real sample, but neither had been taken into account in our simulation.

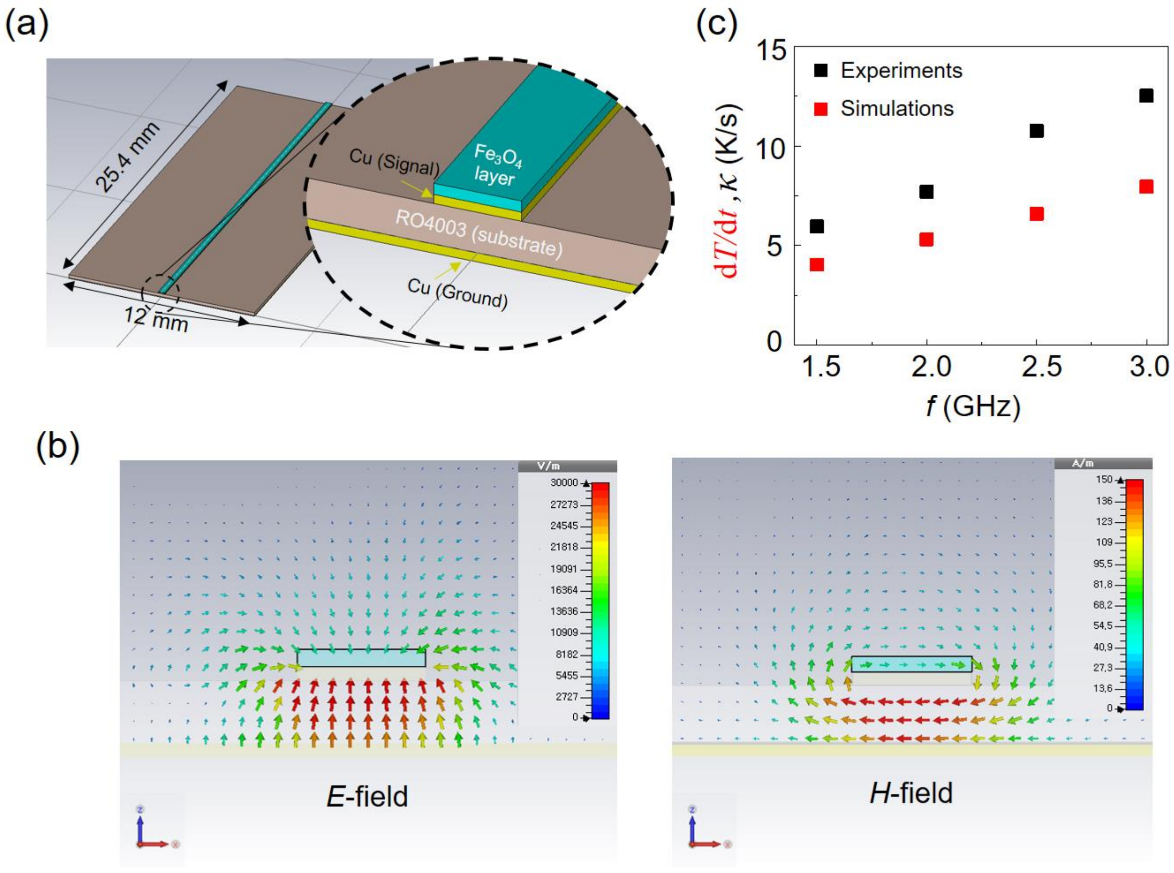

Figure S6. (a) Modeling for electromagnetic simulation of spatial distribution of both electric and magnetic fields. The dimensions are the same as those of the real sample, but the Fe_3O_4 particles were assumed to be a 50 μm-thick continuous film placed on the Cu signal line. (b) Snapshot images of spatial distribution of both electric (E) and magnetic (H) fields for f_{AC} = 3.0 GHz with input power of 1 W. (c) Temperature increment rates obtained from electromagnetic simulations (red squares) and experimental measurements (black squares) versus the AC frequency with input power of 5 W.

Table S1. Material parameters used for electromagnetic simulations

	**Cu**	**RO4003**[20]	**Fe_3O_4**[21,22]
Permittivity (ε_r)	Electrical conductivity<br>$\sigma = 5.96\times10^7$	3.55	2.57
Dielectric Loss<br>tanδ ($=\varepsilon_r''/\varepsilon_r'$)		0.0027	0.3<br>(at 3 GHz)
Density (kg/m^3)	8,930	1,790	5,180
Thermal conductivity<br>(W/K·m)	401	0.64	7
Heat capacity (J/K·kg)	390	900	650

S6. Curves of temperature increment *vs* time according to different external field parameters

A. Temperature variation for cases of f_{AC} = 1.5, 2.0, and 2.5 GHz

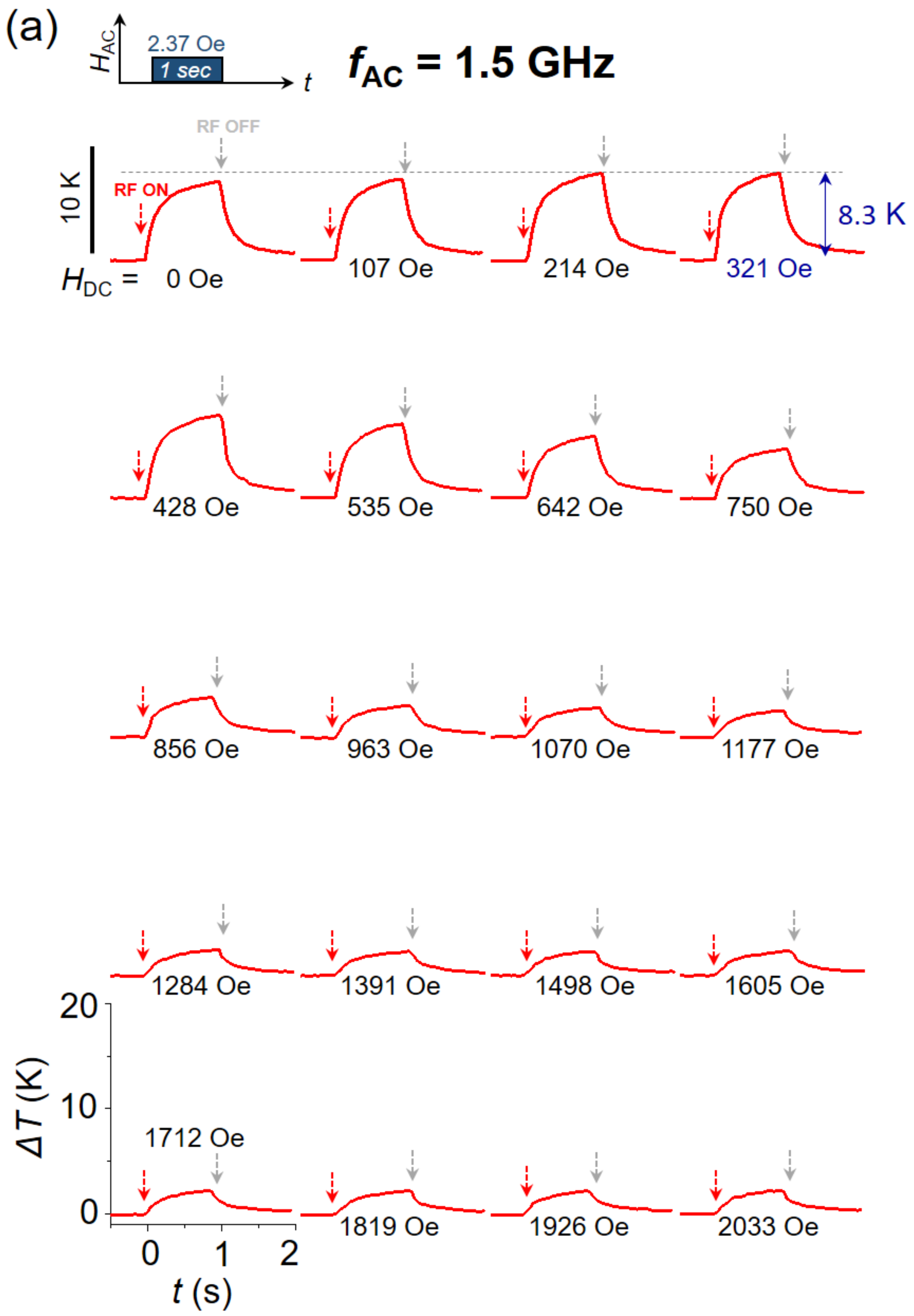

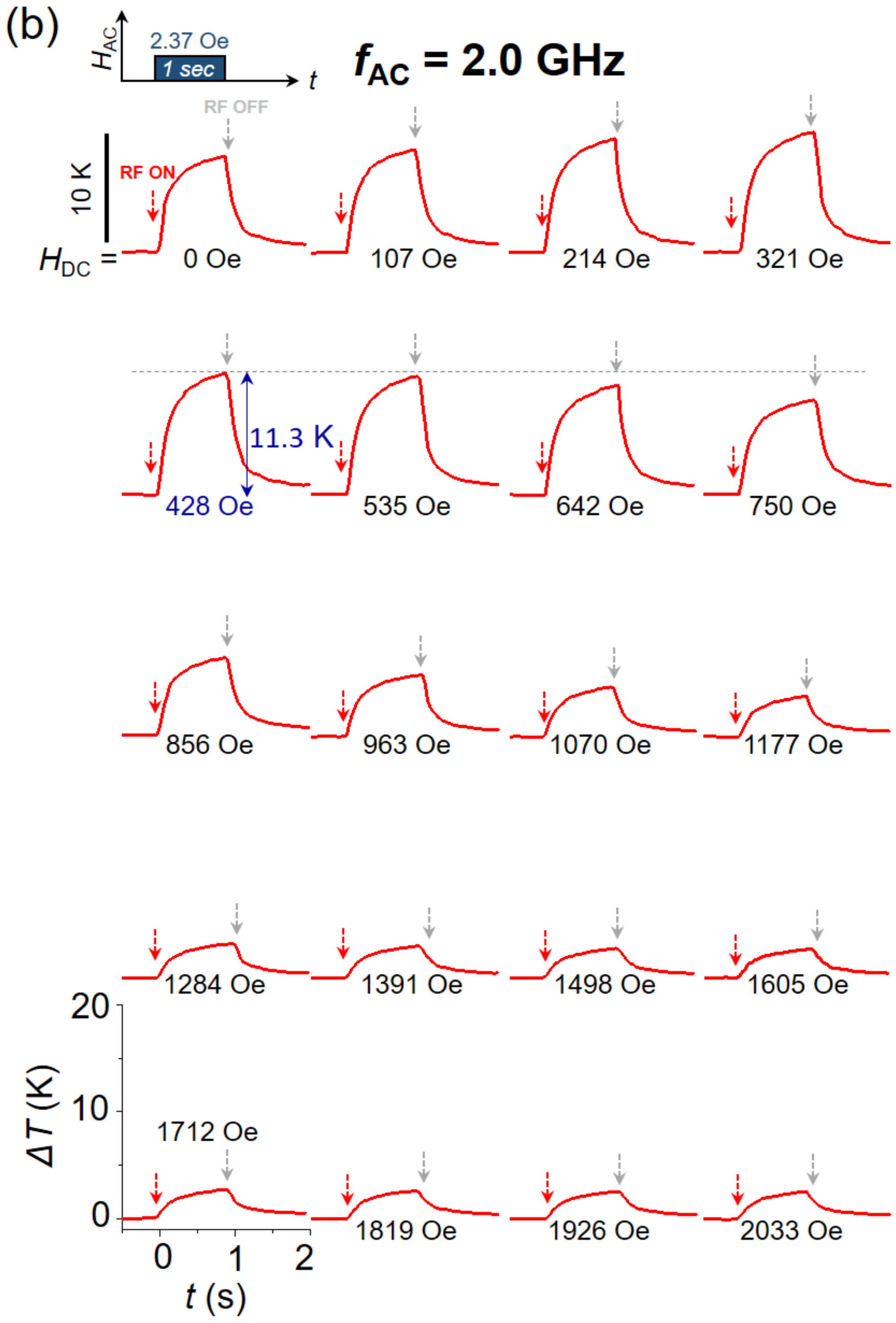
(b)
H_{AC}
2.37 Oe
1 sec
t
f_{AC} = 2.0 GHz
RF OFF
RF ON
10 K
H_{DC} =
0 Oe
107 Oe
214 Oe
321 Oe
11.3 K
428 Oe
535 Oe
642 Oe
750 Oe
856 Oe
963 Oe
1070 Oe
1177 Oe
1284 Oe
1391 Oe
1498 Oe
1605 Oe
20
10
0
ΔT (K)
1712 Oe
1819 Oe
1926 Oe
2033 Oe
0
1
2
t (s)

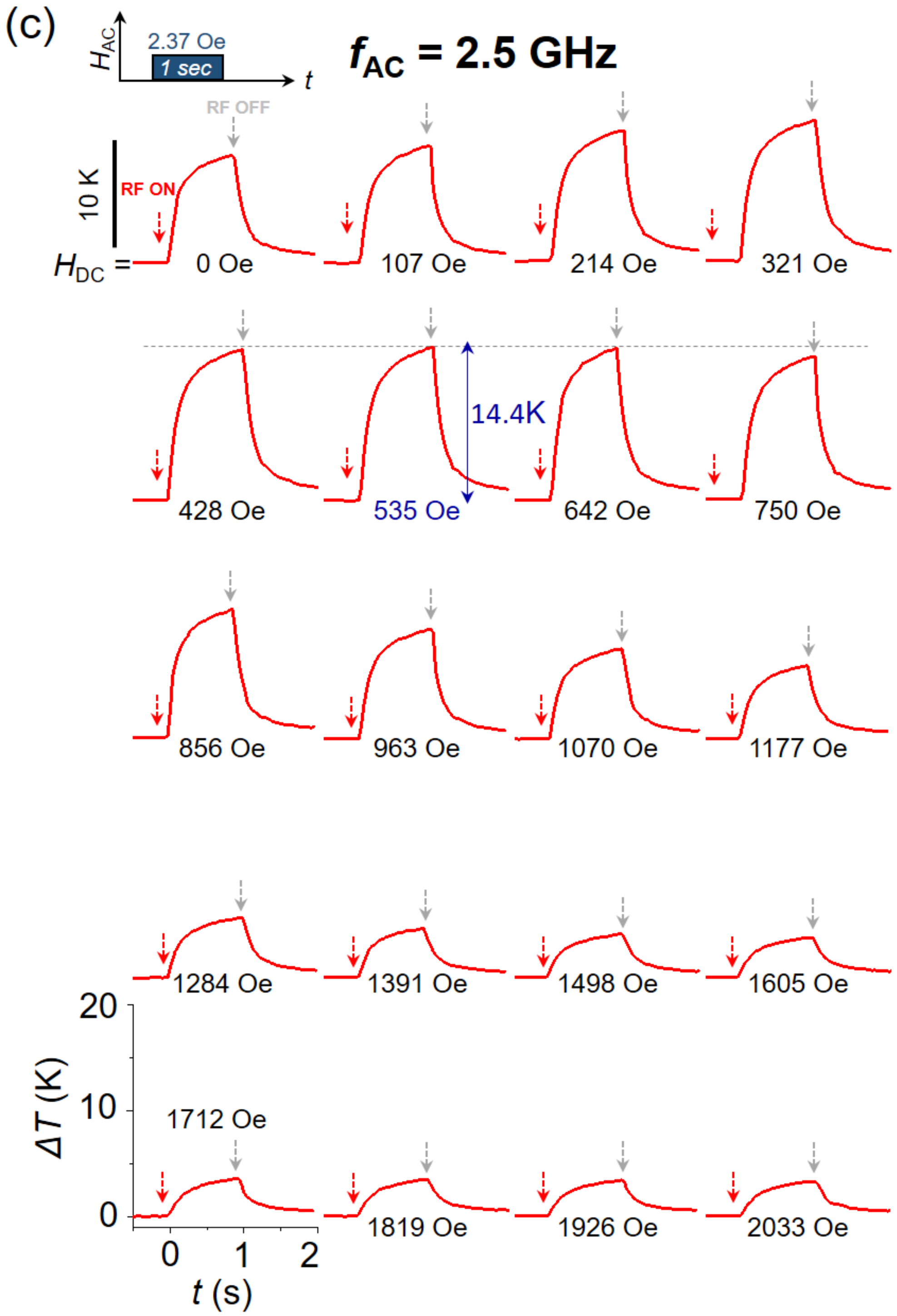

Figure S7(a-c). Temperature variation versus time for different H_{DC} values (H_{DC} = 0 ~ 2033 Oe in steps of 106 Oe) on application of 1 sec-duration pulsed RF magnetic field with H_{AC} = 2.37 and (a) f_{AC} = 1.5 GHz, (b) 2.0 GHz, and (c) 2.5 GHz

B. Temperature *vs* time for different AC field strengths H_{AC}

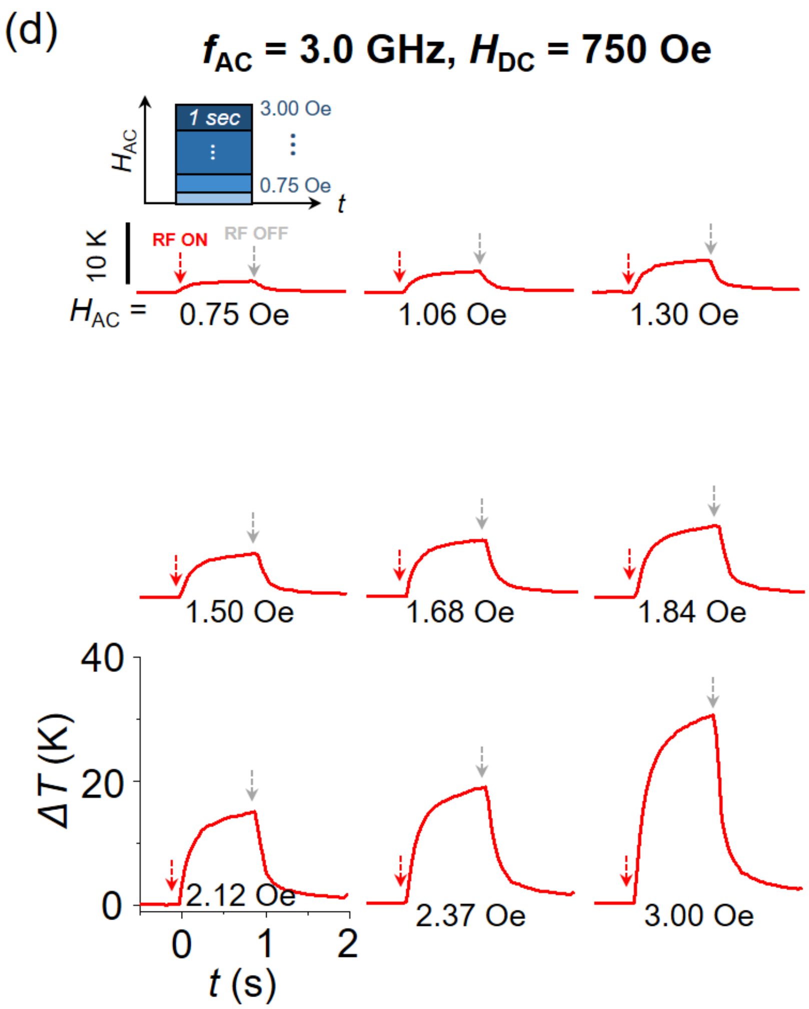

Figure S7(d). Temperature variation versus time for different H_{AC} values (H_{AC} = 0.75 ~ 3.00 Oe) on application of 1-sec-duration pulse of AC magnetic field of f_{AC} = 3.0 GHz at H_{DC} = 750 Oe (resonance condition)

C. Temperature *vs* time for different pulse widths σ of AC magnetic fields

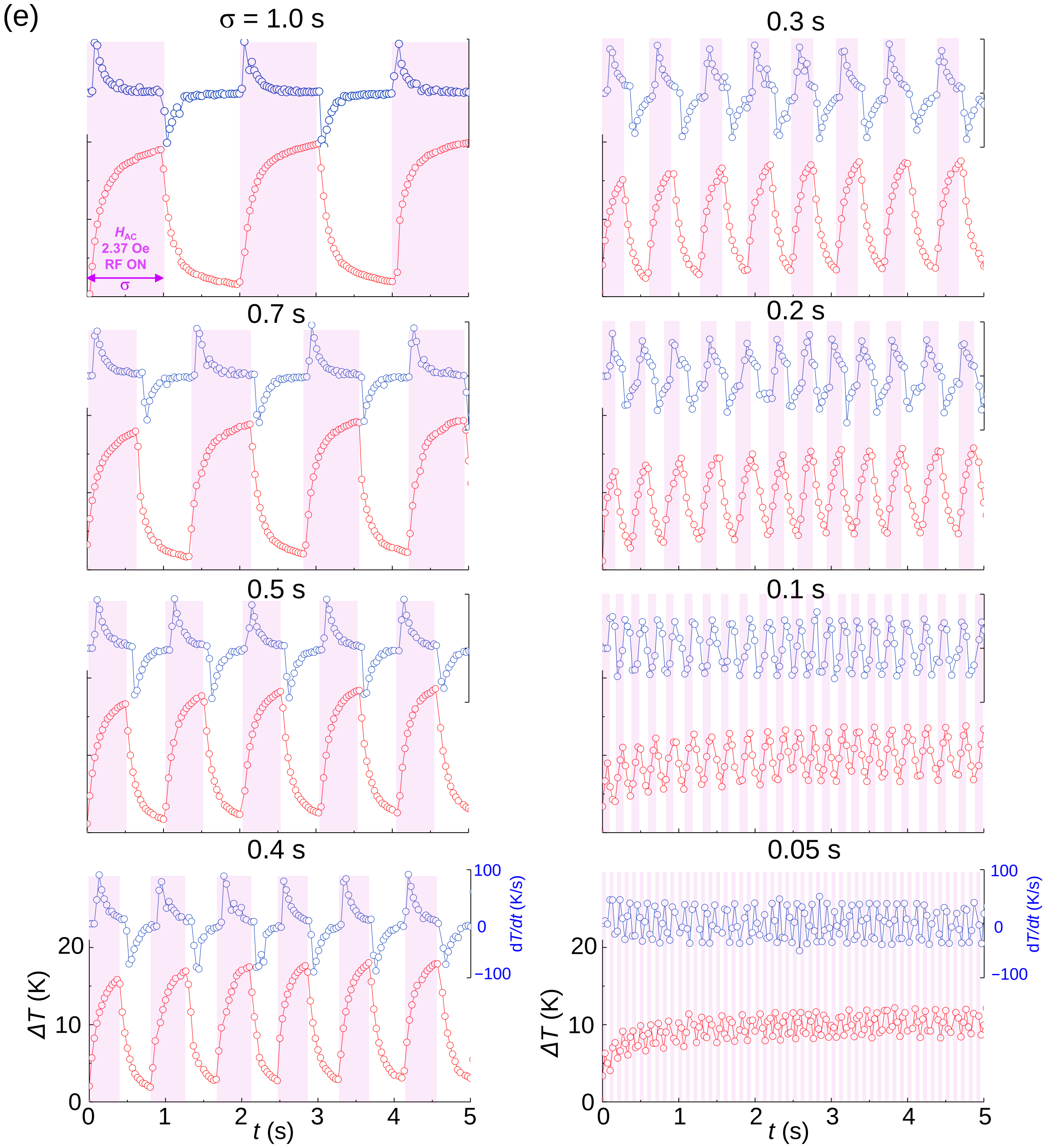

Figure S7(e). Temperature (red symbols) *vs* time along with time-derivative profile (blue symbols) for application of periodic pulses of different pulse widths σ for f_{AC} = 3 GHz and H_{AC} = 2.37 Oe at H_{DC} = 750 Oe (resonance condition)

Supplementary References

1. Jang, J. T. *et al.* Critical Enhancements of MRI Contrast and Hyperthermic Effects by Dopant-Controlled Magnetic Nanoparticles. *Angew. Chem. Int. Edit.* **48**, 1234-1238 (2009).

2. Lee, J. H. *et al.* Artificially engineered magnetic nanoparticles for ultra-sensitive molecular imaging. *Nat. Med*. **13,** 95-99 (2007).

3. Usamentiaga, R. *et al.* Infrared thermography for temperature measurement and non-destructive testing. *Sensors* **14**, 12305-12348 (2014).

4. Bramson, M. A. *Infrared Radiation: A Handbook for Applications* (Plenum, New York, 1968).

5. Hillebrands, B. & Ounadjela, K. (Eds.) *Spin dynamics in confined magnetic structures I*, (Springer, Berlin, 2002).

6. Kim, B., Cho, Y. J., Bhoi, B., Park, S. Y. & Kim, S. K. Hetero-interface effect on Gilbert damping in nonmagnetic metal/permalloy/nonmagnetic metal trilayers. *J. Magn. Magn. Mater.* **465,** 399-405 (2018).

7. Kronmüller, H., Miltat, J. E. & Scheinfein, M.R. (eds). *Handbook of Magnetism and Advanced Magnetic Materials* (John Wiley & Sons Ltd., New York, 2007).

8. Poperechny, I. S. & Raikher, Y. L. Ferromagnetic resonance in uniaxial superparamagnetic particles. *Phys. Rev. B* **93**, 014441 (2016).

9. Kim, S. K. *et al.* Resonantly excited precession motion of three-dimensional vortex core in magnetic nanospheres. *Sci. Rep.* **5**, 11370 (2015).

10. Respaud, M. *et al.* High-frequency ferromagnetic resonance on ultrafine cobalt particles. *Phys. Rev. B* **59** R3934-R3937 (1999).

11. Castel, V., Ben, Y. J. & Brosseau, C. Broadband ferromagnetic resonance measurements in Ni/ZnO and Ni gamma-Fe_2O_3 nanocomposites. *J. Nanomater.* **2007**, 27437 *(*2007).

12. Fannin, P. C., Marin, C. N., Malaescu, I. & Stefu, N. An investigation of the microscopic and macroscopic properties of magnetic fluids. *Physica B* **388**, 87-92 (2007).

13. Marin, C. N. The particle concentration effect on magnetic resonance linewidth for magnetic liquids with chain aggregates. *J. Magn. Magn. Mater.* **250**, 197-202 (2002).

14. Valenzuela, R., Herbst, F. & Ammar, S. Ferromagnetic resonance in Ni-Zn ferrite nanoparticles in different aggregation states. *J. Magn. Magn. Mater.* 324, 3398-3401 (2012).

15. Schrefl, T. & Fidler, J. 3D calculation of magnetization processes in Co/Pt multilayers. *J. Magn. Magn. Mater.* 155, 389-392 (1996).

16. Szalay, A. S. *et al.* Indexing the Sphere with the Hierarchical Triangular Mesh. *arXiv:cs/0701164* (2007).

17. Afremov, L. & Panov, A. Magnetic states and hysteresis properties of small magnetite particles. *Fiz Met Metalloved* **86**, 65-73 (1998).

18. Uesaka, Y., Fukushima, H. & Inaba, N. Accuracy of 45 degrees torque method for obtaining anisotropy constant of 2D random films. *IEEE Trans. Magn.* **35**, 2673-2675 (1999).

19. Poole, C. & Darwazeh, I. *Microwave Active Circuit Analysis and Design* (Academic Press, Cambridge, 2016).

20. Material parameters of RO4003 can be found in www.rogerscorp.com.

21. Guan, P. F., Zhang, X. F. & Guo, J. Assembled Fe_3O_4 nanoparticles on graphene for enhanced electromagnetic wave losses. *Appl. Phys. Lett.* **101**, 153108 (2012).

22. Yin, P. F. *et al.* Facile synthesis and microwave absorption investigation of activated carbon@Fe_3O_4 composites in the low frequency band. *RSC Adv.* **8**, 23048-23057 (2018).
